# Acute Respiratory Distress

**DOI:** 10.5811/cpcem.2018.5.38041

**Published:** 2018-07-16

**Authors:** James Maloy, Nicholas Harrison, Amit Bahl

**Affiliations:** Beaumont Health System, Department of Emergency Medicine, Royal Oak, Michigan

## CASE PRESENTATION

A 24-year-old woman presented to the emergency department by emergency medical services with severe respiratory distress and hypoxia. The patient complained of exertional chest pain and nonproductive cough. Her room air saturation was 65% with improvement to 95% with oxygen supplementation. Her vital signs were a pulse of 110 beats per minute, blood pressure of 140/100 mmHg and a temperature of 36.5 degrees Celsius. Lungs were clear to auscultation, heart was without murmur, and extremities had no edema. Electrocardiogram demonstrated sinus tachycardia with rSR’ pattern, prominent p-waves, and an elevated R:S wave ratio in V1 and V2. Troponin was 0.08 ng/mL, d-dimer was 445 ng/mL, and hemoglobin was 16.4 g/dL. Portable chest radiograph was normal.

**Image 1:**
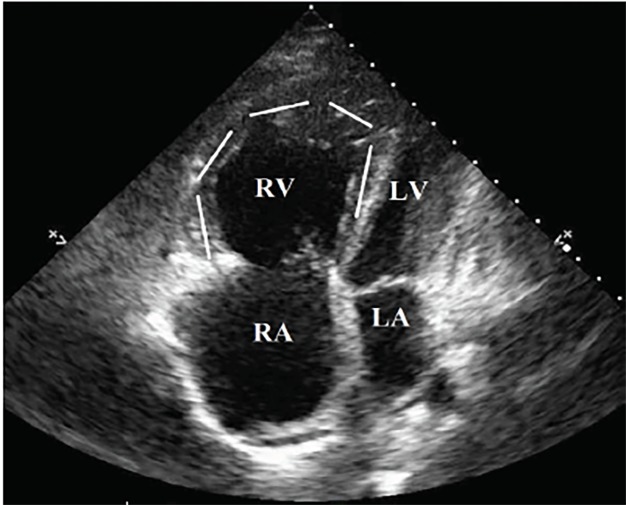
Transthoracic echocardiogram apical view demonstrating severe right ventricular (RV, walls outlined) and right atrial (RA) dilation consistent with right heart strain. *LV,* left ventricle; *LA*, left atrium.

**Image 2:**
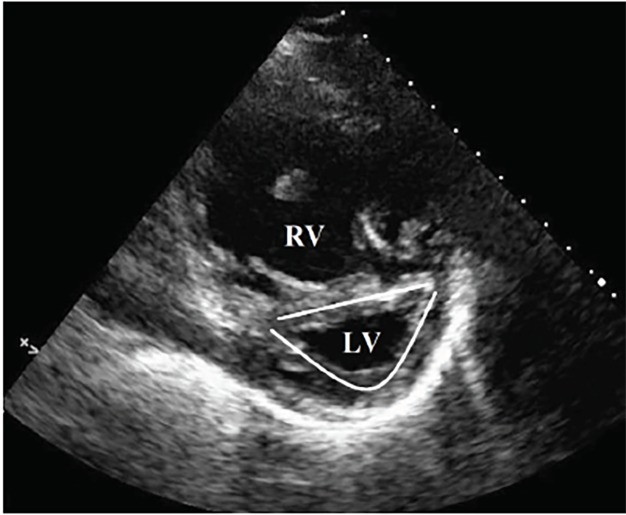
Transthoracic echocardiogram parasternal short view demonstrating enlargement of the right ventricle (RV) with bowing of the interventricular septum (straight line) toward the left ventricle (LV), demonstrating the “D” sign (outlined) of right ventricular overload.

Point-of-care ultrasound (POCUS) demonstrated significant right ventricular dilatation (Image 1) with hypertrophy of the right ventricular myocardium (Image 2). On further questioning, the patient clarified that she had been diagnosed with “pulmonary hypertension” but hadn’t seen a doctor in over a year and was not prescribed any treatment. Subsequent review of outside electronic medical records revealed an echocardiogram performed approximately one year prior to presentation that demonstrated concern for an atrial septal defect.

POCUS revealed significant right ventricular hypertrophy supporting a longstanding disease process. Computed tomography angiography did not reveal any abnormalities. The patient was admitted for hypoxia and pulmonary hypertension. On admission, formal echocardiogram demonstrated concern for atrial septal defect with left-to-right shunt. Two days later, repeat echocardiography with bubble study demonstrated right-to-left shunt across the interatrial septum. The patient rapidly decompensated during the admission, leading to intubation for respiratory distress and then pulseless electrical activity arrest and death despite resuscitation.

## DIAGNOSIS

Eisenmenger syndrome is the process by which a longstanding, left-to-right cardiac shunt, secondary to congenital heart defect, reverses into a cyanotic right-to-left shunt.^1^ The reversal is a result of progressive pulmonary over-circulation, with a subsequent increase of right ventricular pressures over the course of several years. This also gives rise to right ventricular and pulmonary artery hypertrophy. POCUS reveals evidence not only of right heart strain but also significant right ventricular hypertrophy,^2^ helping differentiate the etiology from massive pulmonary embolism. In a patient with desaturation despite high supplemental oxygen and no evidence of lung consolidation, right-to-left cardiac shunt should be considered.

Documented patient informed consent and/or Institutional Review Board approval has been obtained and filed for publication of this case report.

**CPC-EM Capsule**

What do we already know about this clinical entity?

In Eisenmenger syndrome, a right to left intracardiac shunt causes progressive pulmonary hypertension. Once pressures in the right heart exceed those in the left, shunt reversal and severe hypoxia occurs.

What is the major impact of the image(s)?

While right ventricle (RV) dilation and hypoxia often suggests pulmonary embolus, RV hypertrophy should prompt consideration of chronic pulmonary hypertension and alternative diagnoses.

How might this improve emergency medicine practice?

Differentiating acute vs. chronic right heart strain is poorly described, but management of each can be very different. Ultrasound may be useful in distinguishing the two.
